# Parents’ Experiences of Receiving the Initial Positive Newborn Screening (NBS) Result for Cystic Fibrosis and Sickle Cell Disease

**DOI:** 10.1007/s10897-016-9959-4

**Published:** 2016-04-20

**Authors:** Jane Chudleigh, Sarah Buckingham, Jo Dignan, Sandra O’Driscoll, Kemi Johnson, David Rees, Hilary Wyatt, Alison Metcalfe

**Affiliations:** 1 Florence Nightingale Faculty of Nursing & Midwifery, King’s College London, James Clerk Maxwell Building, 57 Waterloo Road, London, SE1 8WA UK; 2 Cystic Fibrosis Paediatric Service, King’s College Hospital NHS Foundation Trust, London, UK; 3 Department of Paediatric Haematology, King’s College Hospital NHS Foundation Trust, London, UK; 4 South East London Sickle Cell and Thalassaemia Centre, Guy’s and St. Thomas’ NHS Foundation Trust, London, UK

**Keywords:** Newborn screening, Cystic fibrosis, Sickle cell disease, Psychosocial

## Abstract

The clinical advantages of the newborn screening programme (NBS) in the UK are well described in the literature. However, there has been little exploration of the psychosocial impact on the family. This study followed the principles of grounded theory to explore parents’ experiences of receiving the initial positive NBS result for their child with cystic fibrosis (CF) or sickle cell disease (SCD). Semi-structured, qualitative interviews were conducted with 22 parents (12 mothers and 10 fathers) whose children had been diagnosed with CF or SCD via NBS and were under the age of 1 year at the time of interview. The main themes that arose from the data were; parents previous knowledge of the condition and the NBS programme, the method of delivery and parental reactions to the result, sharing the results with others, the impact on parental relationships and support strategies. Study conclusions indicate that most parents thought initial positive NBS results should be delivered by a health professional with condition specific knowledge, preferably with both parents present. Genetic counselling needs to include a focus on the impact of NBS results on parental relationships. Careful consideration needs to be given to strategies to support parents of babies who have positive NBS results both in terms of the psychological health and to assist them in sharing the diagnosis.

## Introduction

Newborn Screening (NBS) in the United Kingdom (UK) allows for presymptomatic identification and early initiation of treatment for babies affected by genetic or congenital conditions such as sickle cell disease (SCD) and cystic fibrosis (CF) (UK Newborn Screening Programme Centre 2012).

NBS for SCD was introduced in the UK in 2001 and in 2004, the first antenatal and NBS programme for women and children with SCD and thalassaemia was set up in response to government recommendations (DoH 2000). This was the world’s first antenatal and NBS programme where the results of parental tests were ‘linked’ with those of their baby. The antenatal screening process for SCD varies in the UK with only those mothers in high prevalence areas being automatically offered a blood test for SCD; preferably prior to the 10th week of pregnancy. In areas where haemoglobin diseases are less common, the family origin questionnaire (FOQ) is used to determine which women should be offered antenatal screening (Daniel and Henthorn 2012). In the region in which this study was undertaken, the maternal blood sample is subject to high performance liquid chromatography (HPLC) to identify the presence of haemoglobin variants such as those causing SCD. Following antenatal screening, if the mother is identified as a carrier of SCD, the baby’s father is offered a blood test to identify if he is also a carrier. If both parents are found to be carriers, they should be offered genetic counselling where genetic risk will be discussed.

After birth, parents are invited to have their baby screened as part of the NBS programme. Again, in the region in which this study was undertaken, HPLC followed by isoelectric focusing (IEF) is used to identify haemoglobin variants. These will differentiate between infants who are unaffected (presence of Hb F and Hb A), infants who are affected (Hb F and Hb S in the absence of Hb A) and those who are carriers (presence of Hb F, Hb A and Hb s) (Daniel and Henthorn 2012). Parents are therefore informed of their child’s definitive diagnosis when receiving the NBS result.

This differs from the screening procedure for CF. Currently antenatal screening for CF is not routinely practiced in the UK; parents are not typically aware of their own genetic status during the antenatal period which means those parents who are carriers are not cognisant of the subsequent risk to their unborn child.

NBS for CF has been available throughout the UK since October 2007. The purpose of the CF NBS protocol in the UK is to maximise the detection of affected individuals (those with two disease causing mutations of the CF transmembrane regulator (CFTR) gene) while minimising the detection of unaffected carriers of CF. The current protocol consists of the initial immunoreactive trypsinogen (IRT) assay which is used to identify those infants with elevated levels of IRT which is a sensitive but not specific indicator for CF followed by a one or two stage mutation analysis of the CFTR gene if the IRT value is ≥99.5th centile; commonly referred to as the IRT /DNA protocol. This initial analysis of the blood spot sample taken during NBS is therefore used to identify babies at increased risk of CF and further confirmatory testing (sweat test) is required for a definitive diagnosis (Green et al. 2014).

In the UK, Cystic Fibrosis (CF) affects about 1 in every 2500 babies while Sickle Cell Disease (SCD) affects about 1 in every 2000 babies. In the UK in 2012–13, of over 810,000 babies who were screened for CF, 362 were found to be affected and of over 770,000 babies who were screened for SCD, 323 were found to be affected; almost equal to predicted values (Morgan 2014). Furthermore, approximately 150 and 10,000 babies were found to be healthy carriers of CF and SCD respectively.

The clinical advantages of NBS are well described in the literature; identification of affected children leads to early diagnosis and consequently better health outcomes for the child (Bush 2008; UK Newborn Screening Programme Centre 2012). This enables health professionals (HPs) to provide support to prospective families so they can make informed choices during subsequent pregnancies and before conception in addition to the potential to improve infant health through prompt identification of affected babies. Furthermore, in SCD there is the specific advantage that penicillin prophylaxis can be started before 3 months, which has been shown to reduce morbidity associated with invasive pneumococcal infections (Hirst and Owusu-Ofori 2014).

However, it is important to consider that in the UK, NBS is offered on a voluntary basis. Consequently parents are required to provide informed consent and therefore ‘opt in’ to the screening programme. This is different from other parts of the world such as the United States of America (USA) (where in all but one state) and Canada where an ‘opt out’ approach is taken. In practice, this means parents in the UK should be adequately prepared and informed to participate in the NBS programme. However, research has shown that often parents see NBS as a ‘fait accompli’ and as such do not consider that they have a choice to decline (Nicholls and Southern 2012) which may impact upon their experience when presented with a positive NBS results.

In reality, as can be seen from the incidence data (Morgan 2014), healthy carrier status is the second commonest outcome that parents and HPs will discuss in practice, after normal results. Consequently, there has been a heavy focus on communication of carrier results in the literature which has highlighted that this can lead to initial heightened anxiety in parents (Kai et al. 2009; Ulph et al. 2015) as well as longer term issues related to disclosing the results to the child (Ulph et al. 2014). However, there is a paucity of literature that focusses on the communication needs and experiences of families when an infant receives a positive screening result indicating they are likely to be or are affected by CF or SCD. Factors that influence parents’ experience following the initial delivery of positive NBS results when CF or SCD have been largely overlooked. Moreover, where guidance exists, this has not been evaluated despite literature highlighting parental dissatisfaction with the process of sharing the positive NBS result (Collins et al. 2012; Ulph et al. 2015).

Although the clinical advantages of NBS for CF and SCD are clear, the communication needs of families when a child has been identified as being affected by the condition and any psychosocial impact requires further exploration. Therefore the research question guiding this study was ‘what are parents’ experiences and perceptions of receiving a positive NBS result for their child with CF or SCD?’ The aims were to gain insight into the parents’ experiences, explore the effects of the result on the family, the impact on the parents’ relationships with health professionals and consider alternative ways of sharing positive NBS results with parents.

## Methods

### Design

This was a qualitative study using semi structured interviews and the core principles of grounded theory (Charmaz 2006, 2014; Corbin and Strauss 2008). As very little evidence exists with regard to the experiences of parents receiving the initial positive NBS result when CF or SCD is suspected, this allowed for the gradual emergence of a theory describing their experiences and influential factors.

### Participants

Parents were recruited from one specialist CF and SCD Centre. In line with the principles of grounded theory, purposeful and theoretical sampling were used to explore categories as they emerged as data collection and analysis proceeded. Parents (mothers and fathers) of infants aged <12 months who had been diagnosed with CF or SCD via the NBS programme were invited to participate.

### Inclusion and Exclusion Criteria

Families where both the mother and father were present and agreed to be interviewed were targeted. It has been highlighted in the literature that fathers often feel marginalised during antenatal screening which they reported impeded their ability to support their partner (Williams et al. 2011). Therefore, this study sought to include fathers’ and mothers’ views and experiences of receiving the NBS result. Parents (mothers and fathers) whose babies were born at term without coexisting medical conditions that could impact on family coping mechanisms and whose parents had no history of any prior psychosocial issues that would exclude them from participation were identified by the condition specific Clinical Nurse Specialist (CNS).

### Data Collection and Analysis

Data was analysed using techniques from the grounded theory approach (Charmaz 2006, 2014; Corbin and Strauss 2008). In-depth, semi-structured interviews were undertaken with parents of infants aged <12 months who had received an initial positive NBS screening result for CF or SCD and for CF, those who had undergone further confirmatory testing. An initial interview guide was developed from the literature but was adapted as the study progressed in line with the grounded theory approach. All interviews were tape recorded and transcribed verbatim.

Data collection and analysis were undertaken concurrently in order to explore, deepen and refine future questions as the work progressed (Barroso 2010; Charmaz 2006, 2014; Corbin and Strauss 2008). The first stage of data analysis involved open coding where interview transcripts were analysed in detail (line by line) using an inductive approach to identify initial codes. Key words, phrases and excerpts were assigned codes based on what they represented for example, parents’ initial responses to receiving the positive NBS result. To a large extent, this involved in vivo codes in order to stay as close to the data as possible. Similar codes were then grouped to form categories. Each category was carefully defined to ensure the consistent description of the codes contained within it as data analysis progressed. In order to validate the initial coding process, it is usual when using the grounded theory approach, to utilise an iterative process; checking the interpretation and analysis of the data with research participants. However, due to this being a highly emotive time for parents, it was decided that an iterative process would not be incorporated as it was considered to be unnecessarily distressing for parents to relive their experiences more than once. However, constant comparative methods were used to check data within and between interviews to look for similarities and differences, to ensure the codes and categories were consistent and representative of the data and that analysis continued to stay close to the data. Using the constant comparative method also helped to identify further interview questions to be used in subsequent interviews in order to explore meaning and deepen understanding of concepts as they arose.

The second stage of data analysis, axial coding, involved identifying the relationships between the codes and categories that had emerged during open coding; this often occurred in conjunction with open coding. Both inductive and deductive approaches were used; as mentioned, inductive approaches led to the development of the initial codes and categories which were then reviewed during this stage of data analysis along with related memos to identify relationships and connections between the categories. Deductive approaches were used to ascertain whether the identified relationships and connections were consistent within and between interview transcripts as data collection and analysis progressed.

Finally, selective coding was undertaken which involved finding the core concept (s) that linked the codes and categories identified during open and axial coding. In this way, the initial labels and categories identified during open coding were refined through axial and selective coding until core variables were identified and the theory began to emerge. Constant comparative methods were used to establish comparisons and distinctions between previously generated codes and data from subsequent interview transcripts; parental responses were constantly compared both within and between the CF and SCD groups (Charmaz 2006, 2014; Corbin and Strauss 2008).

Memo writing was used during data collection and all stages of data analysis. Memos similar to field notes were written following each interview and included details regarding the context of the interview. This also encouraged the interviewer to remain reflexive during data collection. During data analysis memos were written to explore and act as a reminder regarding how initial codes were defined, how there were then subsumed into categories and how relationships and connections between them were developed. Reviewing memos during the data analysis process ensured that the analysis remained consistent but also true to the data (Charmaz 2006, 2014; Corbin and Strauss 2008).

### Recruitment

The condition specific CNS informed the Principal Investigator (PI) of the dates of hospital clinic appointments for potential participants. At the appointment, the PI provided parents with the study information and then contacted parents by telephone 1 week later to determine if they would like to be involved and if so, an appointment was made to undertake the interview. Parents were offered a choice of location for the interview for their convenience; home, hospital or place of work. Full consent for the study was sought on the date of the interview after additional questions had been answered and prior to the interview being conducted.

### Ethical Approval

Ethical approval was obtained (# 13/LO/1181) from the Research Ethics Committee (REC) and local research and development department of the participating specialist centre.

## Results

A total of 12 families were included in the study; 12 mothers (5 with a child with CF and 7 with a child with SCD) and 10 fathers (5 each with a child with CF or SCD).

All potentially eligible families identified by the CF CNS were included in the study. Fifteen potentially eligible families were identified by the SCD CNS. Of these, two families declined to participate but chose not to disclose why, one family was subsequently not available to be interviewed and 5 families were excluded as the parents had separated (*n* = 3) or the partner had returned to another country (*n* = 2) and therefore was not available for the interview. Of the remaining seven families, both parents were interviewed in five cases and only the mother was interviewed in the remaining 2 families as their partners subsequently became unavailable after the interview with the mother had been arranged/conducted.

The child’s age at the time of the interview, birth order of the child, interview duration and location and any family history of SCD or CF can be seen in Table 1. All but one interview took place in the family’s home. The results herein are reported under the themes that emerged from the data.

**Table 1:** Interview details for study participants

Child	Age of Child at Interview (months)	Birth Order	Family History of Condition	Duration of interview (minutes)		Location of interview	
				Mother	Father	Mother	Father
SCD							
1	6	1st	No	38	15	Home	Home
2	3	1st	Dad SC, maternal niece SS	43	22	Home	Home
3	5	1st	Maternal brother SS	19	14	Home	Home
4	2	2nd	Dad’s nephew SS	23	27	Home	Home
5	11	4th	Sibling SS	30	16	Home	Work
6	5	1st	Maternal grandmother SS	41	N/A	Home	N/A
7	3	1st	No	17	N/A	Home	N/A
CF							
A	11	1st	2nd cousin	24	34	Home	Work
B	9	1st	No	65	69	Home	Home
C	4	1st	No	50	17	Home	Home
D	5	1st	No	70	23	Home	Home
E	4	1st	Granddad’s cousin’s grandson	33	31	Home	Home

### Theme 1: prior Knowledge of the Condition (CF and SCD) and NBS

Parental knowledge regarding the specific conditions and the NBS programme differed markedly for CF and SCD. Two out of five families with a child with CF had a family history of the condition (Child A and E). One mother was a children’s nurse (Child D) who specialised in diabetes and therefore had clinical experience of working with children with CF. Despite this, prior knowledge of CF was generally limited and this intensified their feelings of anxiety upon receiving the initial positive NBS result.

I didn’t really know what cystic fibrosis was. You see things, bits and pieces, I knew it was something to do with breathing and lungs, but I didn’t know much else.’

Mum of Child E

All but one father remembered receiving information either during pregnancy or shortly after birth about NBS either verbally or in writing. However, parents reported feeling falsely reassured by the HP who provided the information about the likelihood of receiving a positive NBS result or that the information was not relevant to them. Therefore in most instances, they had not read the information.

‘We were told ‘these things are all very rare’.. .’You’ll get a letter through in a few weeks that will say everything is fine’. ‘It’s just a routine test.’

Mum of Child B

No parent recalled giving informed consent for NBS to be undertaken and notably, expressed the view that they felt it was simply a test that was undertaken on all babies.

Of the seven families with a child with SCD, five had a family history of SCD and the remaining two had experience of close friends with the condition. Additionally, one mother worked as a health care assistant in a haemoglobinopathy ward and consequently had first-hand experience of caring for adult patients with SCD. Many parents were able to give a good account of how sickle cell affects red blood cells, the importance of maintaining hydration, taking prescribed medication and the potential impact of infections. One mother expressed finding her prior experience reassuring as she felt she knew what to expect in the future. However, for other parents this led to them viewing SCD quite negatively and fearing for their child’s future.

I’m always worried about his future. Even though I have a positive mind about it, there’s still something that’s saying, ‘oh dear, what?’...I was talking with my husband and I asked him, ‘What if he wants to play football? Will he be able to play football?’ and I had so many questions on my mind. I was like, ‘How’s it going to be? What if he wants to do-,’

Mum of Child 1

In stark contrast to parents of children with CF, parents of children with SCD generally reported feeling well informed about the screening process.

‘I was told why [having blood antenatally]… They told us there’s a 25 % chance we can have this SS baby, AS I think it’s 25 % or about 50 % AA…She had to get my consent to do it [NBS] and I told her it was alright for her to do it…’ Mum of Child 1

Therefore, parents of babies with SCD described being able to make an informed decision regarding their participation in the NBS process as well as an understanding of the possible outcomes and insight into the possible implications positive NBS results for SCD may have.

### Theme 2: Receiving the Initial Positive NBS Result

All families recalled receiving the initial positive NBS results vividly. For the families with children with CF, two mothers received the news on their own (Child A and B). This was viewed negatively and fathers expressed negative emotions related to their inability to support the child’s mother during this time.

‘…one of the things I regret is not being here at the time… That’s something I can’t really change and wish I could, because that’s an experience that no mum should have to go through on their own and I’d like to have been here for that

Dad of Child B

In contrast, for the three families for whom the mother was not alone when she received the positive NBS result for CF, both parents expressed how important it was for them to have someone there to support them both when the result was delivered and immediately afterwards.

Four families of babies with suspected CF received the news, face-to-face from a HP (health visitor (HV), or paediatric respiratory nurse specialist) but this was not viewed positively. This was due to the HVs perceived lack of knowledge and therefore inability to answer parental questions about CF and subsequently, the lack of support the parents felt the HV was able to provide.

‘She [the HV] just dropped the bombshell and then left, but she couldn’t really explain to me exactly what cystic fibrosis was. I did ask [HV], ‘So what is it then? What does that exactly mean?’ and she couldn’t tell me what cystic fibrosis was… I didn’t really feel any support from her [HV], but I suppose it wasn’t easy for her to have given me the news anyway’

Mum of Child B

Furthermore, the perceived lack of knowledge of the HV during this initial meeting had a detrimental effect on future relationships between the parents and the HV. Consequently parents generally favoured the support provided by the CF clinical nurse specialist at the hospital. Given parents general lack of prior knowledge related to CF, the inability of the HP delivering the positive result to parents to provide information, reassurance and answer parental questions was viewed very poorly by parents.

One family received a telephone call from a doctor at the specialist CF centre and reported feeling pleased that they were able to ask questions immediately and have them answered by a professional with condition specific knowledge. However, they expressed the view that they would have preferred this was not conducted over the telephone.

For the families who received the positive NBS result for SCD, three mothers were alone and four were with a family member or friend. However, contrary to the families of infants with suspected CF, none of the parents of infants with SCD commented on the impact of receiving the result alone or with somebody else present.

Of the seven families who received a positive NBS result for SCD, five received a letter or telephone call informing them someone would be visiting them to deliver the results of the screening test and were subsequently given the results face-to-face. Receiving the letter caused parents some degree of anxiety and trepidation.

‘I got a letter saying that someone is going to come and see me. The letter says that she’s not going to be coming with the health visitor. I don’t really understand, but I’m thinking now that I think there’s something wrong’ Mum of Child 2

In contrast to the experience described by the families of babies with CF, parents of babies with SCD spoke very highly of the support and information they received from the specialist SCD community nurse who discussed the initial positive NBS results with them. This was mainly related to her ability to answer parental questions, explain immediate treatment options and provide contact details for any future queries related to SCD.

‘When she [SCD specialist community nurse] came, she was really lovely. She explained everything, she tried to calm us down first before she told us the thing’ Mum of Child 1

### Theme 3: Reactions to the Positive NBS Result

Parents of children with CF and SCD reported feeling a range of emotions when they received the initial positive NBS screening results. This included relief, devastation, guilt, denial, surprise and shock.

‘It hit me like a ton of bricks. I picked [Baby] up and just went next door with her and just, like, cried my eyes out really well if truth be known and, ‘Why us?”

Dad of Child D

Parents expressed the view that their reactions were related to some extent to the fact that although they could recall the ‘heel prick test’ they could not recall explicitly being informed what their child had been screened for. In addition, parents alluded to a sense of false reassurance provided by the HP at the time of screening regarding the likelihood of a positive result.

Despite the excellent knowledge and understanding parents of babies with SCD demonstrated about the screening process and the possible outcomes, they still described feelings of shock, disbelief and guilt when their child was identified as being affected by SCD.

‘At the time I got the result and they knew it was failed for me, I would say I was depressed because it was too much’

Mum of Child 1

### Theme 4: Sharing the Result with Others

Parents of babies with CF shared the initial NBS results with their close family almost immediately and prior to confirmatory testing. For all families of babies with CF, once both parents were aware of the result, they also informed grandparents and siblings. This occurred in all cases within 24 h of receiving the positive result which meant it was before they had been seen at the specialist CF centre and had the diagnosis confirmed. Parents were also willing to share the results with friends and work colleagues although this more frequently occurred after the diagnosis had been confirmed. Sharing the positive result with grandparents and siblings was generally viewed to be upsetting and difficult.

‘It’s not fair on her [grandma] either because she’s going through-, she was really upset and it wasn’t because of [baby]. It was because I was upset. She kept saying to me, you know, ‘This is really upsetting for you.’ She said, ‘That’s what upsets me…’ ‘My sister came over that evening and telling her was quite horrific…telling my sister was the hardest because she was so excited that I was having a baby, so excited and it did break her heart.’

Mum of Child D

The experience of sharing the NBS results with others (not family members) was varied and while some found it therapeutic others did not consider it a pleasant experience. The former stated that the process of telling others could be empowering and also helped to highlight information that they were unaware of so that they could either research CF further or ask the specialist CF team during their next hospital visit.

For those parents who found it unpleasant, this was mainly due to the responses of others which could be viewed as quite insensitive or represent misconceptions about CF. Also, demands on parents to answer other peoples’ questions when they were still coming to terms with the result themselves were found to be challenging.

‘So to begin with, it was quite hard, because they were asking us questions, and we were trying to get our heads around it ourselves and give them information.’…‘It did actually help I think, me and [Dad] a little bit, having to tell other people. It sounds a bit strange, in a way, but because we did have to tell other people what was going on, it helped us to understand it a bit better, I think, because we had to get it clear in our minds, what it was.’

Mum of Child B

‘The ignorance I’ve found a challenge. Going back to work and people not understanding cystic fibrosis, for me was frustrating... There’s a lot of … ignorance in terms of what it is. There are also a number of common misconceptions.’

Dad of Child B

However, on the whole, parents of babies with CF were keen to share the positive NBS results with family and friends. This is in stark contrast to parents of babies with SCD of whom the majority discussed the importance of not disclosing their child’s NBS results with others including family members. For many, this was culturally motivated and stemmed from a fear of the reactions of others and associated social stigma and the perception that recipients of the news would pity them. Comments included fearing that people would look at their child differently, that people would mock them, talk about them and feel sorry for them when the parents preferred not to dwell on the outcome of the NBS result.

I don’t want them [family] to know because the way they’re going to take it. I don’t want people looking at her saying she’s not a healthy child. ‘If someone knows about it then they will feel pity…I can’t really explain…but, I just know that the way they’re going to be looking at her, it’s not going to be like normal child that hasn’t got anything.’

Mum of Child 2

Parents cited examples of their experiences of friends and relatives with SCD when they had lived in or visited Africa. They commented that these experiences often tainted the views of relatives still living in Africa who held a very bleak outlook in relation to those with SCD and in their opinion, did not appreciate the differences between the African and UK health care systems. Parents also described a fear that relatives and friends would hold misconceptions about SCD in relation to life expectancy and the side effects of medications.

She [mother in law] was saying, ‘He’s going to be sick all the time, he’s going to be this, he’s not this.’ All this negative force...she was saying ‘Sickle cell is sickle cell.’...‘Penicillin is going to make his penis go in, he’s not going to perform.’

Mum of Child 1

Parents also expressed concern for their child’s future as a result of sharing their positive NBS results. Their fears particularly focussed on who their child would marry once other people in the community knew given the social stigma surrounding the SCD diagnosis and who would care for their child in the future.

‘I start thinking that how is she going to get married and everything, because people where we come from, seeing people with sickle cell, they will say, ‘Don’t marry her.’ I’ve started thinking that is she going to have a normal life? Will she find someone that she’s going to love, that’s going to take her home?’ Mum of Baby 2

Therefore, the fear of stigmatisation related to the positive NBS result for SCD often lead to parents withholding the information from family or friends or only sharing it on a need to know basis (for example, child care settings and relevant HPs).

### Theme 5: Impact of the Screening Process on Parental Relationships

Receiving the positive NBS result for CF had the potential to impact on parental relationships. Some parents reported difficulties that stemmed from the baby’s mother informing the baby’s father of the positive NBS result and therefore while the health visitor was seen as being the bearer of bad news for the mother, the mother was seen as having a similar role when she had to deliver the news to the father. Parents also commented that they felt that finding out their child had CF had caused arguments between them. For other parents, it made them question their choice of partner and led to feelings of confusion and guilt although they indicated they had not discussed these feelings with their partner.

In the beginning I did feel, like, ‘Oh, why did we get married?’ I started thinking if I’d married someone else, I wouldn’t have had a baby with him [Father], but then I wouldn’t have him [Baby].

Mum of Child C

This effect was also highlighted by parents of babies with SCD. Often, it was the screening process that identified parents as carriers of SCD and some parents stated that had they known prior to having a child with their partner this may have influenced their decisions.

‘He’s [Dad] SC. I didn’t know initially when we started our relationship. We’d already started and it’s very difficult to stop, because, you know, when you love someone… If I knew at the start of the relationship I wouldn’t carry on. But because, I mean, like,- after like three years, that’s when I knew, so then it’s very difficult for me to pull out. ‘Mum of Baby 2

### Theme 6: Future Support Strategies

When parents of babies with CF received the initial positive NBS result, they were unanimously advised by the HP delivering the news not to browse the internet but to wait until they were seen at the specialist centre the following day to have their questions answered. However, all parents used the internet almost immediately to seek information prior to their hospital appointment and found it to be a poor information source that was not helpful.

‘We had a look online… that was quite scary as well, because if you read some of the stuff online, it’s not good and it tells you about life expectancy. That’s one of the first things that comes up and then you start to think, ‘Oh, that’s really bad. You’ve just got to be careful what you look at, because you can end up reading threads from people who have posted things saying, ‘Oh, I lost my daughter aged whatever. This happened. She was really ill in hospital.’

Mum of Child E

Parents found the information sheets available from a CF charity informative and helpful. In addition to this, parents indicated that they would appreciate the opportunity to have contact with other families with a child with CF as they felt they could get first-hand information about the everyday complexities of caring for a child with CF that they may not glean from HPs. Parents were aware of and acknowledged the difficulties of face-to-face contact due to infection risks but still felt some form of contact would be useful, although they felt this information would need to be tailored and timed according to the needs of the individual.

‘ It would be nice to talk to other CF parents just to find out when they give their medication to their child, or how they get round taking stuff out through the day.’

Mum of Child B

Some parents also stated that having been through the experience themselves, they would be willing to be a supporter of other parents going through the NBS process.

Parents of babies with SCD identified that due to the perceived social stigma associated with having a child with SCD, they often felt isolated. They felt that in the future, this stigma should be dispelled to enable parents to meet other parents in the same position and be able to freely discuss their thoughts and fears. They identified the need for social networking opportunities to be established to allow families with children with SCD to meet and share experiences.

‘By talking to the people [who have children with SCD]. At least then…you know, they’ve been through it, so they’ll be able to explain to you, ‘It’s like this, it’s like that,’ so, yes...Then, at least they can give you the honest truth as well...and also speaking to other dads because men always have this issue of talking about things’ Dad of Baby A

Although views about support groups were not always positive but again this related to the perceived social stigma attached to having a child with SCD.

‘I used to go to a supporting group but everybody, you know, if you go there they know your problem. So we’d all be, like, animals at a zoo, just because, you know, because we are different from everyone else.’ Mum of Child E

### Theoretical Framework

**Fig. 1:**
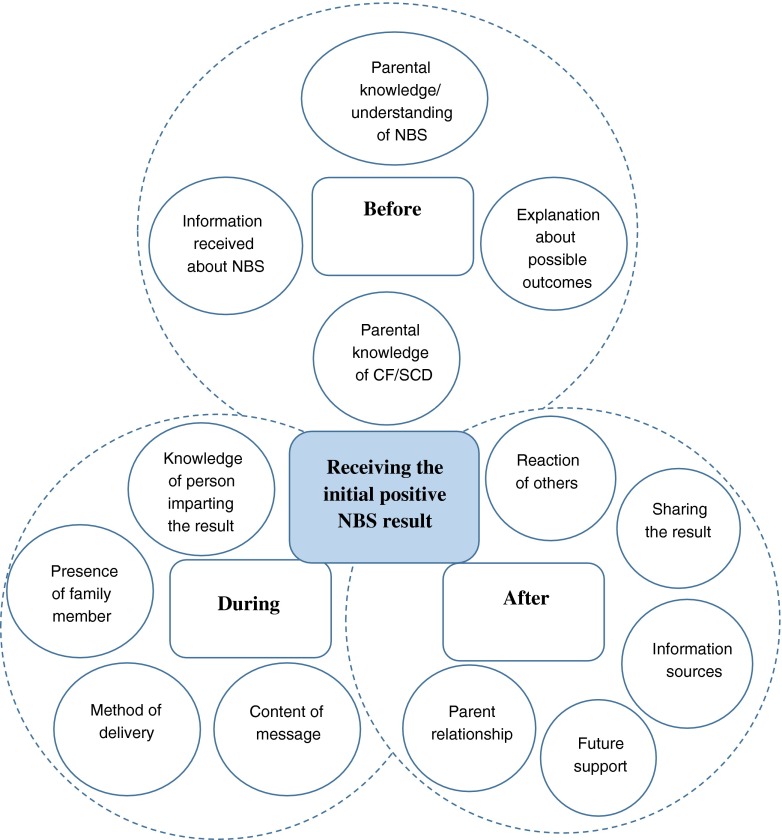
Theoretical Framework exploring factors that impact on parents when receiving a positive NBS result for CF

Using the principal themes that emerged from the study, a grounded theory was developed as depicted in Fig. 1 to describe the factors surrounding the NBS process and their potential impact on the family. There are complex interactions between factors that occur before, during and after the initial positive NBS results are delivered to parents that influence their experience of this process. From the outset, parents need to understand the NBS process, what is being tested for and the likely outcomes as this can impact on their response when they receive the positive NBS result. When they receive the result, this needs to be delivered by someone with knowledge of the specific condition and the content of their message needs to portray this confidently to the parents to secure future relationships between the parent and HP. Ideally, the result should not be given to a parent on their own, if possible both parents should be present. Whether or not the result should be given face-to-face or over the telephone prior to a person with condition specific knowledge visiting the family, needs to be explored further and may be dependent on how parents are informed about the conditions antenatally. Further support for parents to enable them to confidently share the result with family members and others while minimising associated trauma also needs to be explored so that appropriate strategies specifically targeting this can be developed.

## Discussion

In the present study, there were clear similarities and disparities between families with children with CF and SCD in relation to their experience of receiving the initial positive NBS result. Similar to previous findings (Parker et al. 2007), the HP and method used to deliver the results varied widely, particularly for parents of babies with CF. In the UK the structure of the NBS programmes for CF and SCD differ with the former having no antenatal screening pathway while the latter does. The findings from this study highlight that providing antenatal screening for SCD raised parents’ awareness and knowledge of the condition and the screening process itself which helped them to understand their risk of a positive NBS result. This is supported by findings from previous studies which found that parents were less surprised about their child’s positive NBS result due to having a family history of the condition (Salm et al. 2012) or parents who had prior awareness of carrier status or the possibility of a carrier result (Ulph et al. 2015) assimilated the information about their child’s positive NBS result more readily. In contrast, in the present study, parents of infants with CF felt largely unprepared to receive the news that their child was suspected of having CF due to a perceived lack of information received in the antenatal and immediate newborn period. Parents of babies with CF deemed the information they were provided with prior to NBS to be insufficient or inaccurate and indeed in some cases falsely reassuring. This questions whether or not parents in this study truly gave informed consent for their child to undergo NBS. This is similar to the findings of a previous study conducted in the UK (Nicholls and Southern 2012) and suggests that the content and methods used to deliver information about NBS to parents during the antenatal period and at the actual time of testing needs to be improved to ensure consent is informed.

Following the initial positive NBS result for CF parents are informed that their child is suspected of having CF but further confirmatory testing is required prior to a definitive diagnosis being made. This is in contrast with the positive SCD result which is considered diagnostic. Therefore parents of infants with suspected CF continue to experience a period of uncertainty and ambiguity after receiving the initial positive NBS result while parents of infants with SCD do not. This may also contribute to the differing parental responses to receiving the positive NBS result for each condition.

Parents of babies with SCD found the specialist SCD community nurse reassuring and helpful when providing the NBS results to them. In contrast, parents of children with CF who received the result from a non-CF specialist such as the HV found this resulted in a negative experience. The potential for communication of the results of NBS for CF to either alleviate or exacerbate parents’ responses to positive NBS results and therefore the importance of the HP being knowledgeable has been recognised previously (Salm et al. 2012). This is important since in the present study, distrust in the HVs knowledge base at this early stage in their child’s life for parents of babies with CF, had a deleterious effect on their longer-term relationship with their HV. This is also supported in the previous study (Salm et al. 2012) which found that the approaches used by HPs to deliver positive NBS results had a profound effect on subsequent relationships between the parent and the HP.

Negative experiences related to receiving the NBS result could impact on parental opinions of screening processes per se which may affect screening decisions for future children or other family members. A previous study found that general attitudes towards healthcare staff and the healthcare system play a significant role in determining attitudes towards screening and in turn the quality of decision made: with positive attitudes towards the components of the healthcare system associated with more positive attitudes towards screening (Nicholls and Southern 2014). Finally, parents of infants with CF were keen that the results should not be given to a parent on their own and that there should be someone else, preferably their partner present at this time. Therefore, it is clear that management of the NBS process requires attention and that perhaps inequalities between the services offered for the current conditions screened for need to be addressed. This includes consideration for the provision of antenatal screening for CF and the use of specialists with condition specific knowledge to deliver the initial NBS results to parents preferably when they are together. Due to the fact that CF and SCD differ in that SCD is very common in some areas, particularly London, and very rare in others, whereas CF is evenly distributed across the country, it may be more feasible to train specialist screening nurses who are responsible for the delivery of positive NBS results for all screened conditions. However, the disparities in experiences do present an argument for having condition specific models for the communication of positive NBS results rather than a ‘one size fits all’ approach. Previous work has highlighted condition specific preferences with regard to communication channels for delivery of positive NBS results (Salm et al. 2012).

The present study demonstrated that for parents of babies with CF and SCD, the positive NBS result was unexpected and caused them some degree of distress. For parents of babies with SCD, even though they demonstrated an awareness of the risks to their unborn child following antenatal screening of parents, they clung on to the hope that their child would either be a carrier or unaffected. It is important to consider here that NBS allows for the diagnosis and early treatment of children with CF and SCD prior to them developing any symptoms and therefore appearing obviously ‘unwell’. Therefore, parental shock and disbelief could be akin to a grief response. Certainly, the emotions parents describe such as denial, sadness, surprise and disbelief have been previously described as responses to loss (Kübler-Ross 1969) and are supported by previous studies that have explored parental reactions to positive NBS results (Asplin 2008; Buchbinder and Timmermans 2012; DeLuca et al. 2011). Therefore, it could be argued that parents were grieving for the ‘healthy’ child they thought they had and their dreams and aspirations for their child’s future. Furthermore, it was clear that, particularly for parents of babies with SCD, even during these early stages of the family’s journey, parents had given careful consideration to the impact of the diagnosis on their child’s future. Therefore further psychological support at this time is imperative. Previous work supports this for SCD as genetic counselling for families of children identified as having an abnormal haemoglobin trait, was beneficial in terms of answering questions and reducing parental anxiety (Kladny et al. 2011). For CF, a tailored, family centred model for genetic counselling has been proposed following work that indicated that mismatched counselling could exacerbate already high level of anxiety in parents following their child’s positive NBS results (Tluczek et al. 2011).

Receiving the positive NBS result for both CF and SCD had the potential to impact on parental relationships. For parents of babies with CF this included arguments between couples which has also been evident in previous studies (Tluczek et al. 2011; Ulph et al. 2015). Also, a sense of regret related to having a baby with someone who was also a carrier for the specific condition, albeit unknowingly. This is an important finding and suggests that more attention needs to be given to couple counselling to help address these issues. Indeed, the findings of previous work with families of children identified as having an abnormal haemoglobin trait supports this as genetic counselling was found to facilitates dialog between partners about the result and any perceived attribution of blame (Kladny et al. 2011). These differing responses and needs also support the use of a tailored approach to genetic counselling as previously advocated (Tluczek et al. 2011).

There were marked differences between parents of babies with CF and SCD and their willingness to share the positive NBS diagnosis. Parents of babies with CF shared the results widely and even described it as being therapeutic while parents of babies with SCD were reticent to share the result. For the latter, this was mainly a result of the perceived social stigma associated with the positive NBS result for SCD. Indeed, a study conducted in Kenya (Marsh et al. 2011) found that mothers of children with SCD were particularly vulnerable to stigmatisation within families and this was certainly expressed by one of the mothers in the present study (Mum of Baby 1) whose mother-in-law expressed a desire for the mother and father to separate due to them both being carriers. Interestingly, in this study (Marsh et al. 2011), the potential, nature and form of stigmatisation were focussed around the blame and discrimination effects of having a child with SCD and it was concluded that effective communication and management could help to dispel this. Again in the present study, parents’ lack of desire to share their child’s NBS result with others was heavily focussed on perceived misconceptions, some of these being culturally based. Further education in the UK addressing commonly held misconceptions and beliefs regarding SCD could help to dispel some of the social stigma expressed in the present study and allow parents to confidently share their child’s positive NBS result for SCD with others without fear of stigmatisation.

The use of parental support groups was consistently identified by parents of babies with CF and SCD in the present study as being of potential benefit. For both groups, they felt this would help with every day queries and common parenting issues that may not require the advice of a HP. Additionally for parents of babies with SCD, it was felt that support groups may also help to dispel perceived stigma. Due to issues of infection risk for the babies with CF and stigmatisation for families of babies with SCD, it may be necessary to think of alternatives to face-to-face meetings such as the use of online forums and telephone support. This could help to alleviate some of the isolation both sets of parents experienced albeit for different reasons. These findings are also consistent with the findings of a previous study which found that parents of children who had received a positive NBS for congenital hypothyroidism, CF and CF carrier also wanted to meet parents of children with the same NBS result (Salm et al. 2012).

### Study Limitations

This study was conducted with a small sample of parents of babies from one CF and SCD specialist centre therefore the result may not be generalizable to the whole population. However, the use of purposeful and theoretical sampling (in line with a grounded theory approach) meant that the views of a diverse study population were included.

An iterative process was not incorporated into the data collection process as it was thought this may have been unnecessarily distressing for parents. Although this could have influenced the validity of the identified themes, it was considered that constant comparison between parental responses both within and between the two condition groups mitigated against this.

For all parents of babies with CF, the child was their first. This means parents did not have any experience of having a child who had not had a positive NBS result. However, as this study did not explicitly aim to explore the difference between the experiences of parents receiving a positive or negative NBS result, this was not felt to impact on the findings.

For two families with babies with SCD, only the mother was interviewed. It was hoped that in all cases both mothers and fathers would be interviewed to get both perspectives but in these two instances the fathers’ unavailability for interview only came to light during the interview with the mother. However, no new themes emerged exclusively from the interview transcripts from these mothers and therefore this was not considered to have biased the results.

### Practice Implications

The present study has highlighted the importance of the person communicating the initial positive NBS result having condition specific knowledge and therefore the ability to answer parental questions. This could help to alleviate undue anxiety that has been highlighted in previous studies when this role in undertaken by professionals who do not have specific condition or genetic knowledge.

The present study has also indicated that addressing questions related to a potential child’s future in relation to a SCD diagnosis may be of benefit to some families to alleviate undue anxiety.

Effective communication and SCD management is vital to help reduce perceived stigmatisation associated with having a child with SCD.

The encouragement of tailored genetic counselling for all couples following positive NBS results for CF and SCD is important as the present study has highlighted that the result can impact on couples in many ways and that it would be helpful if these were addressed individually.

### Research Recommendations

Research needs to be conducted to clarify which methods of delivering positive NBS results to parents are most effective in terms of helping parents cope with the diagnosis and supporting them in their discussions with family and friends to reduce the social isolation and stigma associated with these genetic conditions.

## Conclusion

There is no doubt that the NBS programme for CF and SCD in the UK has revolutionised the care for children who are found to be affected by these conditions. The psychosocial impact of having an apparently well child diagnosed with a life altering condition and the impact on the family were shown to be overlooked in this study cohort.

Disparities between the NBS process for CF and SCD need to be addressed to ensure an equitable service. The importance of having a HP with specialist knowledge relating to the specific conditions imparting positive NBS results cannot be over emphasised. Peer support strategies need to be explored to address the lack of non-medical support currently available to parents of children with CF and SCD in the early stage of their journey. Strategies to address perceived misconceptions for CF and SCD and social stigma for SCD following NBS could help to provide further support for families in this situation.
